# Currarino syndrome and microcephaly due to a rare 7q36.2 microdeletion: a case report

**DOI:** 10.1186/s13052-018-0500-2

**Published:** 2018-05-25

**Authors:** Lucia Cococcioni, Susanna Paccagnini, Elena Pozzi, Luigina Spaccini, Elisa Cattaneo, Serena Redaelli, Francesca Crosti, Gian Vincenzo Zuccotti

**Affiliations:** 1 Pediatric Department, “V. Buzzi” Children’s Hospital, University of Milan, Via Castelvetro 32, 20154 Milan, Italy; 2 Genetic Service, Department of Obstetrics and Gynecology, “V. Buzzi” Children’s Hospital, University of Milan, Milan, Italy; 3 School of Medicine and Surgery, University Milano-Bicocca, Monza, Italy; 4 Medical Genetic Laboratory, Clinical Pathology Department, S. Gerardo Hospital, Monza, Italy

**Keywords:** Currarino syndrome, Constipation, 7q36 microdeletion

## Abstract

**Background:**

Currarino syndrome is a rare condition characterized by presacral mass, anorectal malformation and sacral dysgenesis.

**Case presentation:**

We report the case of a child that presented chronic constipation, encopresis and mycrocephaly. The characteristics were initially compatible with a case of functional constipation and a therapy with polyethylene glycol was prescribed. After a year, because of poor response, a plain abdominal X-ray was performed, detecting sacrum abnormalities. Finally, a CGH-array analysis was performed and a form of Currarino Syndrome caused by a rare 7q36 microdeletion, was diagnosed.

**Conclusion:**

Occult spinal dysraphism should be suspected in case of poor polyethylene glycol responder constipation, even when evident sacral abnormalities on the physical examination are not detected.

## Background

Currarino syndrome (CS) is a rare congenital malformation characterized by the triad: presacral mass, anorectal malformation and sacral dysgenesis. Several incomplete forms of CS with variable phenotypes are described, since the only mandatory clinical feature for CS diagnosis is the sacral anomaly [1]. The syndrome was first described in 1981 and approximately 300 cases have been documented in the literature [2]. It is caused by an abnormal separation of the neuroectoderm from the endoderm [3]. It is inherited in an autosomal dominant manner and it is caused by haploinsufficiency of the motor neuron and pancreas homeobox 1 (*MNX1*) gene on chromosome 7q36 [4]. *MNX*1 mutations are detected in about 50% of affected individuals, reaching almost 90% in familial cases [3]. CS phenotype may include intractable constipation, bowel obstruction, urinary retention, incontinence, frequent urinary tract infections, weakness, sensory loss and many others, but more than 33% of affected children are asymptomatic [2]. We report the case of a child with CS features combined with microcephaly who has a 7q36 microdeletion.

## Case presentation

**Fig. 1:**
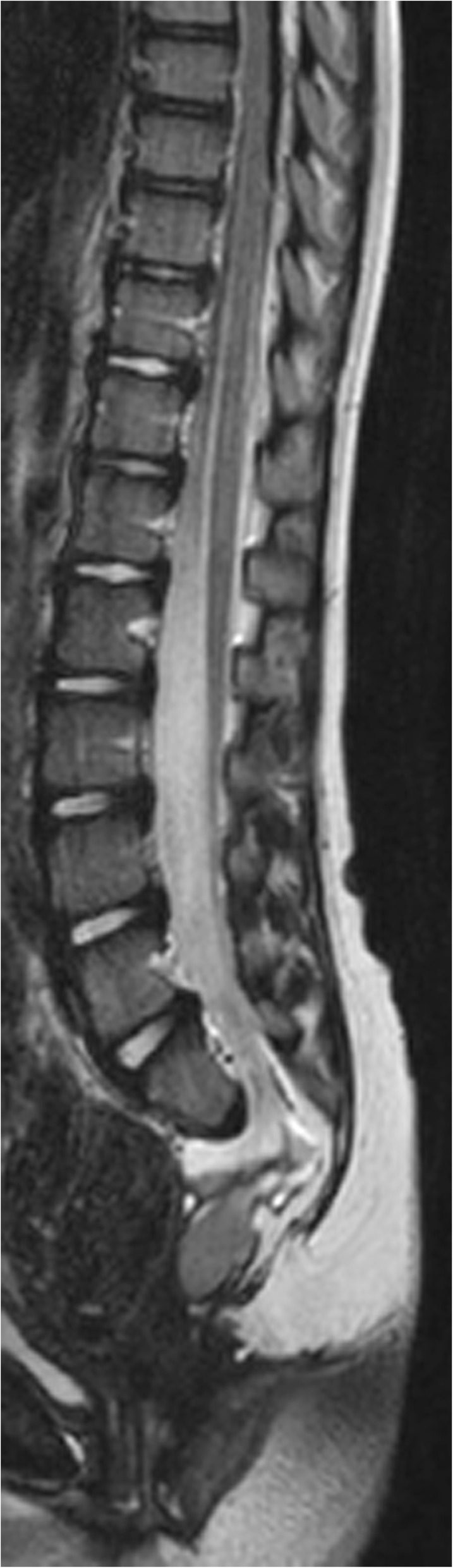
Sagittal t2- weighted Lumbosacral MRI

**Fig. 2:**
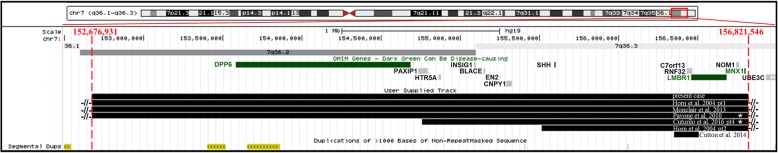
Mapping of the deletion in 7q36.2-7q36.3 for our patient and comparison with literature [4–8]. Schematic representation of common overlapping deleted region (our case breakpoints are in red). * = two patients described by Pavone et al. [7] and patient 1 described by Cuturilo et al. [4] present a duplication more centromeric and consecutive to the deletion (not shown in the figure)

A 3-year-old female child has been evaluated at our gastroenterological service for persisting constipation and encopresis. The symptoms began when the patient was 18 months old. She was the only child born from non-consanguineous healthy Italian parents. The pregnancy was regular and she was born via vaginal delivery after the 38th week of gestation. Neonatal weight was 3250 g (50°-75° percentile), length 47 cm (10–25° percentile), head circumference 33 cm (25–50° percentile). Apgar score was 9–10 -10. Clinical evaluation at birth was normal. Her perinatal period was uneventful. When the child was 26 months old, microcephaly was detected. Cerebral magnetic resonance imaging (MRI) and electroencephalogram (EEG) were performed and both exams resulted normal. Her physical and cognitive development was unremarkable. The family history was negative for genetic diseases. On physical examination, abdominal distention and microcephaly (head circumference 45 cm, < 3° percentile) were the main characteristics detected. The growth was regular, the facial appearance, as well as the sacral and anal regions, were normal. Functional constipation was initially supposed, so toilet training combined with polyethylene glycol (PEG) treatment was prescribed. During the clinical follow-up, constipation and encopresis persisted despite adequate compliance to therapy, so the patient was revaluated at our gastroenterological service. Normal anal sphincter tone and soft stools in rectum were detected at digital rectal examination. Blood tests, particularly thyroid function, celiac disease screening and electrolytes, resulted within normal ranges. On plain abdominal x-ray (AXR) fecal impaction was confirmed and partial sacral agenesis and lumbar dextro scoliosis were identified. A lumbosacral MRI was performed and the following features were recorded: “sickle – shaped” sacrum (S2-S5 and coccygeal vertebrae agenesis) with preserved S1, anterior meningocele, pre sacral teratoma, terminal cord lipoma, low-lying conus medullaris and tethered cord (Fig. 1). To complete the diagnostic evaluation, the urinary tract was studied with abdominal ultrasound and urodynamic test. Both tests resulted normal. As the CS was suspected, *MNX*1 gene sequencing was performed, but no mutation was detected. Nevertheless, the CGH-array analysis identified a de novo 4.15 Mb deletion of 7q36.2q36.3 region, including *MNX*1 and *SHH* genes that are responsible for CS phenotype and microcephaly (Fig. 2) [4–8]. The deletion involves also the *DPP6, PAXIP1, HTR5A, EN2* and *LMBR1* genes. Because of the association between *DPP6* mutations and cardiac arrhythmias, a complete cardiological assessment (electrocardiogram, echocardiogram) was performed, but all these exams resulted in normal range. In addition, it has been reported that the *DPP6* gene appears to play a major role in the regulation of proliferation and migration of neurons [9]. Loss-of-function mutations in *DPP6* are also associated with microcephaly and intellectual disability. In the last neurological evaluation of our patient, which was performed when the child was 6 years old, mild behavioral abnormalities have been documented. In the literature the *EN2* gene has been considered to be a susceptibility gene for autism. Furthermore, mutations of *LMBR1* has been associated with polydactyly. However, both of these conditions were not present in our patient. To the best of our knowledge, deletions of *PAXIP1* and *HTR5A* are not associated with any clinical abnormalities.

The patient underwent a neurosurgical operation with the indication of cord untethering. After the surgical treatment, constipation improved and encopresis regressed. Clinical follow-up is still ongoing.

## Discussion

According to last ESPGHAN and NASPGHAN guidelines about constipation, the history and the clinical characteristics of our patient (regular growth, normal physical examination, the absence of alarm signs and symptoms) were initially compatible with a diagnosis of functional constipation [10]. This disorder is a very common problem during the childhood (estimated prevalence of 3% worldwide) and it is responsible for more than 95% of cases of constipation in healthy children older than 1 year of age [11]. Infrequent bowel movements, hard small feces, difficult or painful evacuation of large-diameter stools and fecal incontinence are frequent complaints in children affected by this condition [12].

In our patient, despite toilet training and oral medication, the symptoms persisted. We decided to complete the diagnostic process with blood tests and abdominal X ray to investigate the presence of fecal impaction and to study the vertebral spine. The radiological test detected an occult spinal dysraphism with partial sacral agenesis. These findings are typical of the Caudal Regression Syndrome (CRS), also known as caudal displasia or Sacral Agenesis Syndrome. This is a very rare disorder of the distal spinal segments with estimated incidence of 0.1–0.25 cases per 10,000 normal pregnancies. In addition, a 200 times increased incidence has recorded in infants of diabetic mothers, where it occurs in about 1 in 350 infants [13]. The syndrome is the result of a neural tube defect that occurs before 28 days of gestation. While the exact etiology and pathogenetic mechanism are poorly understood, several factors such as maternal diabetes, vascular hypoperfusion, drugs (i.e. minoxidil, thripethoprim/sulphamethoxazole, chemicals, fat solvents and appetite suppressants) and genetic predisposition (i.e. *VANGL1* gene mutation) have been suggested to have a causative role [14]. The CRS may range from absent coccyx as an isolated finding without neurological sequelae, to sacral or lumbosacral agenesis. Additional complications of the genitourinary, gastrointestinal and respiratory systems may occur. The CRS spectrum can be categorized in different types. In fact, several classifications are available based on the amount of the sacrum remaining, the characteristics of the articulation between the spine and pelvis and the presence or absence of myelomenigocele [15–17]. The CS, historically known as “Currarino’s triad”, is considered a form of CRS characterized by the classic triad of presacral mass, sacral bone defect and anorectal malformation. CS was suspected in our patient due to the sacrum peculiar morphology and the presacral mass, shown by the spine MRI. The specific sacral anomaly characterized by partial sacral agenesis with intact first sacral vertebra (“sickle-shaped sacrum”), is typical of this syndrome, although other associated congenital malformations and/or developmental delay have been reported. CS phenotypic expression is variable, some patient being asymptomatic while others presenting the complete triad. Autosomal dominant mutations in the *MNX1* gene cause nearly all familial and 30% of sporadic cases. Furthermore, deletions of the 7q36 region have been reported in association with CS [3, 18]. In addition, sporadic cases of CS with a partial duplication of the long arm of the chromosome 3 but no *MNX1* mutation have been reported in the literature [19]. Despite the extreme phenotipic variability observed in patients carrying the same mutation, as well as the incomplete penetrance in familiar cases, recent studies have tried to demonstrate an association between mutation type and severity of the phenotype [20, 21].

The analysis of Merello E. et al. [20], that reported a series of CS patients carrying only intragenic mutations, has failed to establish a clear genotype-phenotype correlation. On the other hand, a more recent multicentre Italian study has reported that the presence of a *MNX1* anomaly, such as an intragenic mutation or a deletion, was associated to a more severe CS phenotype compared to cases in which no mutation was identified [21]. Moreover, according to Cuturilo G. et al. [4], intragenic mutations of *MNX1* are observed more frequently in patients with the classic Currarino triad and regular growth, normal intellect and facial appearance. Instead, CS phenotypes characterized by growth delay and/or facial dimorphism and/or intellectual disability are often due to deletions of long arm of chromosome 7 containing *MNX1* [4]. Indeed, *MNX1* gene seems to be the main responsible factor for the expression and severity of the CS triad, while the associated anomalies appear to be determined by contiguous genes [21].

We performed *MNX1* sequencing first, but it resulted normal. Considering the co-occurrence of a CS-like phenotype and microcephaly reported in previous patients [7], we performed the CGH-array analysis. A microdeletion, i.e. a relative small deletion involving less than 5 Mb of a chromosomal segment, including *MNX1* and *SHH* genes, responsible for the patient’s phenotype was identified by the last test. To the best of our knowledge, this is one of the few reported cases with features of an incomplete form of CS and microcephaly due to a 7q36 microdeletion. As well as our case, patients with deletion involving the same region (Fig. 2) has shown the elements of Currarino triad and microcephaly as main features. However, the previous cases have reported also growth retardation, facial dismorphies, brain abnormalities and other anomalies such as urogenital malformation [4–8]. These characteristics were not present in our patient, underlining the extreme phenotypic variability of CS, as reported in the literature.

## Conclusion

Occult spinal dysraphism should be suspected in case of poor PEG responder constipation, even when evident sacral abnormalities on the physical examination are not detected. In this case, instrumental tests (i.e. abdominal x-ray/MRI) should be performed. Particularly abdominal x-ray is a first level test, cost-effective and easy to perform which can give useful information about rachis morphology.

When a CS phenotype is associated with malformations not included in the classic Currarino’s triad, the CGH-array is recommended as the test of choice, since the presence of a 7q36 deletion is more likely. If microdeletions or microduplications are not identified, *MNX1* gene sequencing should be considered.

## Abbreviations

AXR: Abdominal x ray
CS: Currarino syndrome
EEG: Electroencephalogram
MRI: Magnetic resonance imaging
